# Moral judgement among university students in Ica: A view from the perspective of Lawrence Kohlberg

**DOI:** 10.12688/f1000research.125433.1

**Published:** 2022-12-05

**Authors:** Rosmery Sabina Pozo Enciso, Oscar Arbieto Mamani, Miguel Gerardo Mendoza Vargas

**Affiliations:** 1 Universidad Autónoma de Ica, Perú, Ica, Ica, 11701, Peru; 2 Universidad Nacional Micaela Bastidas de Apurímac, Abancay, Apurímac, 03001, Peru; 3 Universidad La Salle, Arequipa, Arequipa, 04011, Peru

**Keywords:** Moral judgement, L. Kholberg, university students, moral development

## Abstract

**Background:** The aim of this study was to identify moral judgement at the preconventional level, the conventional level, and the post-conventional level in university students in the tenth semester in Ica, in 2022.

**Method:** The research methodology was descriptive-observational, quantitative and cross-sectional. The population consisted of university students in the tenth semester and the sample consisted of 157 university students. A survey was used as a data collection tool and a questionnaire was used to measure the stages of moral judgement according to Lawrence Kholberg.

**Results:** It was found that 12.75% of the study sample was in the instructional relativism stage, 23.10% were in interpersonal agreement, 35.76% were in social order and authority, 11.95% were in social contract and finally 3.80% were in universal ethical principles.

**Conclusion:** It was concluded and identified that the stages of moral judgement among the study sample indicate that interpersonal agreement, social order and authority are the most developed among university students.

## Introduction

When discussing moral judgement, it is essential to talk about “ethics”. This term refers to the fact that a person's behavior within society is regulated by rules. From childhood, human beings are immersed in socializing agents such as the family, the school, the environment, among others ( Mendizabal *et al.,* 2021). Thus, from an early age, children learn that there are both good and bad behaviors. All these opinions forged based on the different experiences and external agents about the moral problems they experience, and the arguments used in favor of these judgements constitute the development of the cognitive behavior of moral judgement ( Noguera, 2018).

As they pass through stages such as adolescence, youth and adulthood, individuals form their own identity, and become consciously aware of assuming and respecting norms and values. Lawrence Kohlberg (1973) points out that the person will learn and move up in the process of moral judgement in accordance with cognitive development. That is, as the person passes through various stages, he or she will find himself or herself at a different moral level, which will modify his or her reasoning ( Mendizabal *et al.,* 2021).

Kohlberg (1969) considers that the functioning of moral judgement is organized in cognitive structures from which all types of moral reasoning derive, which is based on universal and abstract principles, therefore, moral judgement is an evaluation and justification of prescriptive values versus what is good and bad ( Goenaga, Lopera & Villada, 2021).

Kohlberg organized the theory of moral development on the basis of three levels, pre-conventional, conventional and post-conventional. These are characterized by moral problems which, in turn, are divided into two stages representing the individual's criteria for behavior ( Cruz Puerto, 2020). The first level, the preconventional level, indicates that people are not guided by society's norms but only by concrete consequences; at the conventional level, the person adheres to society's norms and strives to avoid being punished; finally, at the postconventional level, the person reasons based on ethical principles beyond society's norms ( Sandoval, Villega & Vega, 2019).

At the beginning of university life, cognitive development is reinforced by teachers, who must contribute to the development of students' moral judgement as part of a comprehensive education ( Mendizabal *et al.,* 2021). Although programs already include ethics and moral issues in their curricula to guide them during their professional practice, the results are mostly imperceptible. Therefore, it is important that based on a diagnosis of the student's reasoning and moral development, the current educational purposes are evaluated and improved ( Contreras *et al.*, 2020).

Moral development has been considered an essential objective in education systems, as it is a progressive and gradual process that requires a combination of academic training, practical skills, and moral competences to be developed ( Ranjbar *et al.*, 2017). This is vital as previous studies show that there is an “ethical erosion” in higher education ( Reyes *et al.*, 2021), therefore, educational commitment must be addressed in the long term, where a theme of values, democracy, commitment, and justice is instilled as an aspect of lifelong learning ( Sandoval, Villega & Vega, 2019).

We must remember that educational institutions are an important partner in the moral development of individuals and society, facilitating change not only at the personal level, but also contributing to the community and society. Citizens must be educated in democratic values, where they exercise their rights in a responsible manner and fulfil their obligations in solidarity ( Pedraza & Pérez, 2019).

In view of this scenario, it was proposed as a general objective to identify and describe the stages of moral judgment among university students in the tenth semester in the district of Ica, in 2022 in a way that allows to demonstrate and respond to the problem that mobilized the study, that is, how are the levels of moral judgment in university students in the tenth semester in Ica in 2022?

For which the following specific objectives were set out: to identify moral judgement at the preconventional level, the conventional level, and the post-conventional level in university students in the tenth semester in Ica, in 2022.

## Methods

### Study design

The deductive research approach was used, because a general topic was analyzed and delimited to the particular and descriptive ( Ñaupas *et al*., 2018).

The study design was quantitative, considering the use of numerical scales for the development of results, non-experimental-observational, because the study was not conducted in a laboratory and the researcher did not intervene in the variables, and cross-sectional ( Hernández-Sampieri & Mendoza, 2018).

### Participants

The population consisted of university students in the tenth cycle of the administrative sciences career from different universities in the district of Ica, Peru. The type of sampling was non-probabilistic, according to Hernández-Sampieri & Mendoza (2018), indicating that this is done to a “subgroup of the population in which the choice of the elements does not depend on probability but on the characteristics of the research” and in turn by convenience, which, according to Valderrama (2014), indicates that the types of sampling by convenience or intentional “is characterized by a deliberate effort to obtain representative samples through the inclusion in the sample of typical groups”.

Given the above in the population, a total study sample of 157 university students (men and women), who were in the tenth cycle of the administrative sciences career, was considered, this being a representative number within the study. The total use of data from the proposed sample was made, not discarding, segregating or excluding any particular data.

### Instruments

The technique used was a survey and the instrument was the questionnaire called “Defining Issues Test” (DIT) which was developed by Rest (1979) and then translated into Spanish by Pérez (1997) as “Cuestionario de Problemas Sociomorales”. Then, Palacios (2003) made a manual called “El uso informatizado del cuestionario de problemas sociomorales (DIT) del Rest” and it was validated by Huanccollucho (2017) with a Cronbach's Alpha reliability of 0.70. The questionnaire was based on the theory of Lawrence Kohlberg (1973), which proposes to examine the moral judgement of a person in each period throughout his or her life and how this may evolve. The full questionnaire can be found in the Extended data.

Kholberg proposes six stages of moral judgement divided into three different levels of development ( Table 1).

**Table 1.** Stages of moral judgement.

Categories	Moral Judgement Stage
Preconventional	Obedience and punishment
	Self-interest
Conventional	Interpersonal accord and conformity
	Authority and maintaining social order
Postconventional	Social contract
	Universal ethical principles

The instrument is divided into six different stories (see Table 2), which are called “dilemmas”, where a short story on a specific topic is presented. Then, twelve items are posed, including questions and statements, per story.

**Table 2.** Stories and items.

Dilemmas	Questions and/or statements
Story 1: Henry and the medicine	12 items
Story 2: The occupation of the students	12 items
Story 3: The escaped prisoner	12 items
Story 4: The doctor's dilemma	12 items
Story 5: Mr. Gómez	12 items
Story 6: The magazine	12 items

At the end of each story, an importance chart is displayed (see Table 3), where the respondent must place the items that he/she considers most important, where 1 is the most important item, according to the student's criteria, and 4 is the item that generates the least importance for the person or the person he/she considers most important.

**Table 3.** Table of importance.

Rank in order of importance				
	1	2	3	4
N° de item				

Table 3 describes what was used to measure the level and development of moral judgement of each university respondent.

### Procedures

The questionnaire survey was carried out using the Google Forms ^TM^ platform, which was distributed to each student in the tenth semester of the administrative sciences course, including instructions for correct completion. All this was done virtually, due to the wave of COVID-19 infection in Peru. The duration of data collection was from June 7 to June 14, 2022.

The statistical software SPSS version 25 was used for data processing, where the reliability assessment (Cronbach's alpha) of the instrument applied was carried out, obtaining a value of 0.702, and the Microsoft Excel 2019 program was used for all the data processing carried out to create tables with the results found. Regarding the presentation of the results of the article, this was done in a descriptive manner, which allowed us to identify the prevalence of the indicators of the moral judgement variable in the study sample. The data processing was carried out from June 15 to June 30, 2022.

### Ethical approval

The project was approved by the president of the ethics committee of the Universidad Autónoma de Ica by means of certificate CO-001-16-2022/CE issued on 15 February 2022.

### Consent

The participants in this study were students between the ages of 22 and 25, who were of legal age for the Peruvian state, and they did not need parental consent to complete the survey. Each of them received a consent form which disclosed all information related to the development of the questionnaire, which they were required to read and sign and then return to the researchers, meaning that written informed consent was obtained from all participants. The anonymity of their participation was also guaranteed and that the answers given would be used exclusively for research purposes, all in compliance with ethical guidelines.

## Results

The preconventional level of the study sample was analyzed, where a development of instrumental relativism moral judgement of 12.75% is evident in the university students in their tenth semester of the administrative sciences (see Table 4). This result is exclusive of the evaluation of developmental stage II, because the test does not evaluate stage I.

**Table 4.** Moral judgement at the preconventional level.

Stage of moral judgement	N	%
Obedience and punishment	-	0.0
Self-interest	157	12.75
Total	157	12.75

N=number of participants.

We analyzed the conventional level where we find a development of moral judgement of interpersonal concordance of 23.10% and social order and authority of 35.76% in the university students in their tenth semester of the administrative sciences corresponding to development stage III and IV respectively (see Table 5).

**Table 5.** Moral judgement at the conventional level.

Stage of moral judgement	N	%
Interpersonal accord and conformity	157	23.10%
Authority and maintaining social order	157	35.76%
Total	157	58.86%

N=number of participants.

The post-conventional level was analyzed, showing a development of moral judgement of social contract of 11.95% and universal ethical principles of 3.80% in the university students in their tenth semester of the administrative sciences corresponding to developmental stage V and VI respectively, determining and reaching a post-conventional level of 15.75% among the study sample (see Table 6).

**Table 6.** Moral judgement at the post-conventional level.

Stage of moral judgement	N	%
Social contract	157	11.95%
Universal ethical principles	157	3.80%
Total	157	15.75%

N=number of participants.

Table 7 shows the A and M scores, which show the reliability of the data, and whether respondents have lied or had a bias when giving an answer. The scores should be less than 14%. If this is achieved, it means that the responses are truthful and do not need to be invalidated.

**Table 7.** Reliability of responses.

Stage of moral judgement	N	%
A	157	6.90%
M	157	5.70
Total	157	12.60%

N=number of participants.

**Figure 1.**
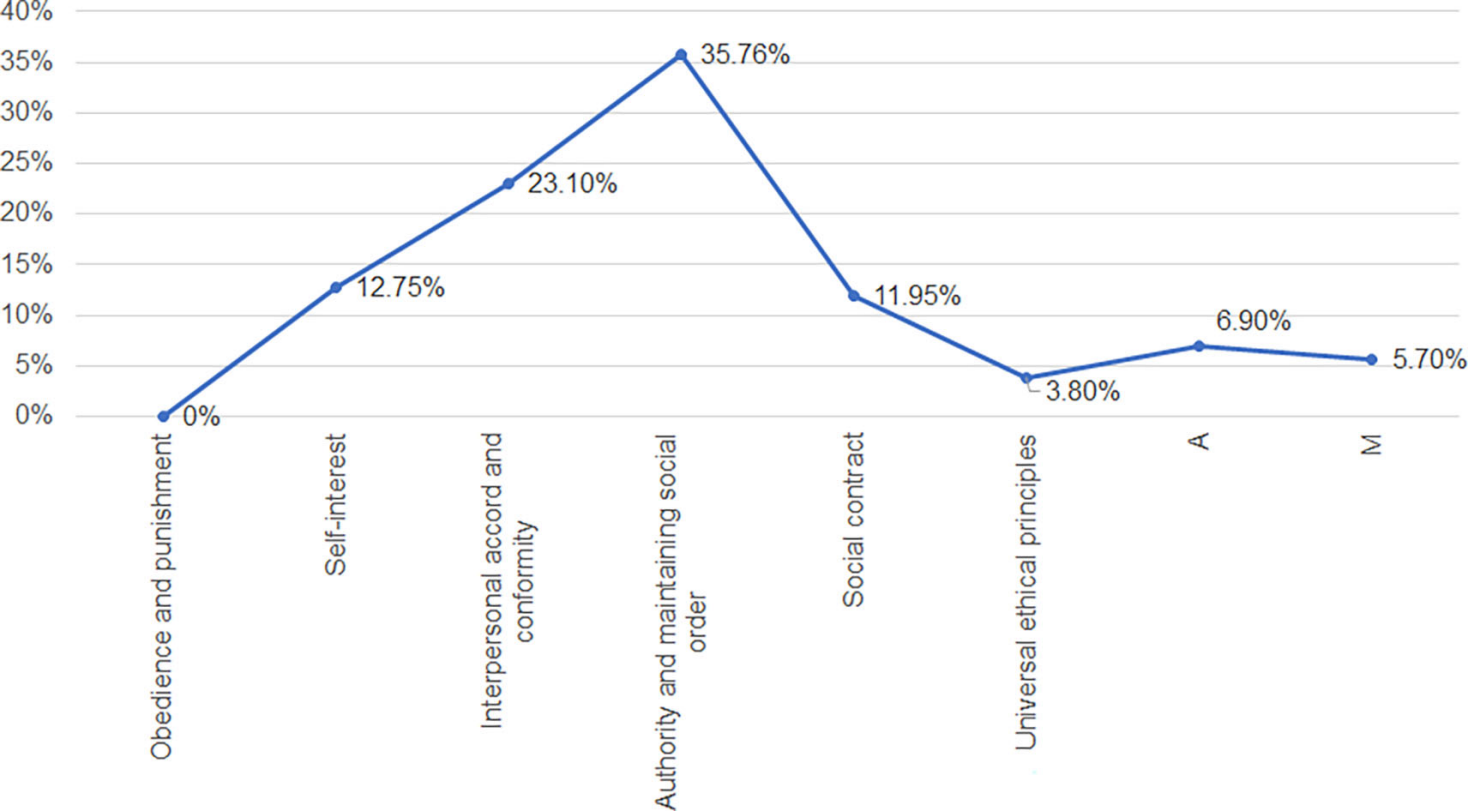
Moral judgement stage profile among the sample: General.

In reference to Figure 1, it is evident that the responses both A (6.90%) and M (5.70%) are less than 14%, so it takes as reliable all the responses collected by the students of the study site. Taking this into account, it is determined that the most predominant moral judgement among the students is the conventional one; being part of this the interpersonal agreement (23.10%) and social order and authority (35.76%): stage III and IV, indicating that the judgement goes with having good behaviors and behaving according to how others expect them to behave, also that they are governed by the regulations and laws determined and that they do not intend to infringe them intentionally, unless they are forced to break some law. Secondly, there is the post-conventional stage; being part of this the social contract (11.95%) and universal ethical principles (3.80%). This group has a judgement empathy, knowing and being aware that all people have different points of view, values, ways of life and so on. They are always being respectful of this, but not preventing that if they see or are in a situation in which they see that the basic and universal principles of a person are being affected; they will put these principles above anything else. And finally, there is the preconventional stage, being part of this the punishment-obedience (0.0%) and instrumental relativism (12.75%), this type of stage would indicate that people act only out of interest and when they know that they will get something beneficial for themselves and for the rest of the people involved, being fairness and justice their predominant ones.

## Discussion and conclusion

The general objective was to identify and describe the stages of moral judgement in university students in the tenth semester in the district of Ica, 2022. Kohlberg (1992) indicates that this stage is based on the individual's sense of morality and is linked to the personal and social relationships that the individual presents. Jaime (2019) found that his study sample also had a prevalence of conventional moral reasoning, with 68.8%, even though the studies were carried out in different educational institutions, time (year of data collection) and academic level of the students; it is evident that the conventional type of judgement is the most deeply rooted within the student in the southern and central area of the Peruvian state.

The specific objectives were to identify moral judgement at the pre-conventional, conventional, and post-conventional levels in university students in the tenth semester in Ica. The results show a development of moral judgement in the preconventional stage, specifically, in the instrumental relativism stage with 12.75%. Kohlberg (1992) indicates that at this level moral sense and judgement is assumed in such a way that norms are accepted if they favor one's own interests. This is because the individual aims to do what satisfies his or her interests, considering it right that others also pursue theirs. The authority figures come from parents or teachers, and minors qualify their actions by their consequences. Regarding the second specific objective, a development of moral judgement of inter-personal agreement of 23.10% and social order and authority of 35.76% was identified, with a total of 58.86% of conventional level and finally, regarding the third specific objective, a development of moral judgement of social contract of 11.95% and universal ethical principles of 3.80% was identified, with a total post-conventional level of 15.75%. Kohlberg (1992) indicates that at this level the meaning of ethics is defined in terms of more abstract principles and values. The person believes that some regulations are unjust and could be changed or repealed. Huanccollucho (2017) concluded that in the preconventional level he obtained a total value of 11.6%, a total conventional level of 44.9% and a total post-conventional level of 21.6%, where it is evident that, as in the present study, the antecedent shows a higher prevalence in stages II and III (conventional).

In all the cases reviewed, it is common for the populations and samples studied to be situated at the conventional level. This means that nowadays young people have become aware of abiding by the common rules of coexistence as well as recognizing that all individuals have interests that may not necessarily be the same. It follows that fairness is relative, as it is linked to personal interests, and that an exchange with others is necessary to ensure that one's own interests are satisfied.

It is important that, in the creation of scenarios for moral development, the educational and teacher's responsibility involves creating a type of conflict that facilitates the development of models of thinking in their students, because, as Kohlberg indicates, models of thinking are not taught, whereas, on the contrary, moral reasoning is self-generated in the environmental exchange and changes progressively.

The main limitation is the collection of data in a single period and not longitudinally to be able to observe how the stages change according to the knowledge and maturity of each person. Therefore, it is suggested that when replicating the studies, they can be carried out under the type of research mentioned above and thus observe the evolution of moral judgement.

It was identified that the stages of moral judgement among the study sample indicate that interpersonal agreement (23.10%); and social order and authority (35.76%) are the most developed, instrumental relativism (12.75%) is the least developed and social contract (11.95%) and universal ethical principles (3.80%) are the stages that tend to develop in the future among university students in the tenth semester in the district of Ica.

Regarding the specific objectives, it was possible to identify that the preconventional level was 12.75%, the conventional level was 58.86% and finally the post-conventional level was 15.75% among university students.

## Data Availability

Zenodo. Moral judgement in university students in Ica: a view from the perspective of L. Kohlberg Vr.4. https://zenodo.org/record/7199915#.Y0Nc-nZBy3A ( Pozo Enciso *et al.*, 2022). This project contains the following underlying data:
• SPSS – Moral judgement database.csv • SPSS – Moral judgement database.sav • MORAL JUDGEMENT DATA BASE.xlsx • MORAL JUDGEMENT DATA KEY.txt • MORAL JUDGEMENT PART 2.xlsx Zenodo. Moral judgement in university students in Ica: a view from the perspective of L. Kohlberg Vr.4. https://zenodo.org/record/7199915#.Y0Nc-nZBy3A ( Pozo Enciso *et al.*, 2022). This project contains the following extended data:
•
Figure 1 - Moral judgement stage profile among the sample: General • Social-moral problems questionnaire.docx Data are available under the terms of the Creative Commons Attribution 4.0 International license (CC-BY 4.0).
