# Moral judgement among university students in Ica: A view from the perspective of Lawrence Kohlberg

**DOI:** 10.12688/f1000research.125433.2

**Published:** 2023-05-09

**Authors:** Rosmery Sabina Pozo Enciso, Oscar Arbieto Mamani, Miguel Gerardo Mendoza Vargas

**Affiliations:** 1 Universidad Autónoma de Ica, Perú, Ica, Ica, 11701, Peru; 2 Universidad Nacional Micaela Bastidas de Apurímac, Abancay, Apurímac, 03001, Peru; 3 Universidad La Salle, Arequipa, Arequipa, 04011, Peru

**Keywords:** Moral judgement, L. Kholberg, university students, moral development

## Abstract

**Background:** The aim of this study was to identify moral judgement at the preconventional level, the conventional level, and the post-conventional level in university students in the tenth semester in Ica, in 2022.

**Method:** The research methodology was descriptive-observational, quantitative and cross-sectional. The population consisted of university students in the tenth semester and the sample consisted of 157 university students. A survey was used as a data collection tool and a questionnaire was used to measure the stages of moral judgement according to Lawrence Kholberg.

**Results:** It was found that 12.75% of the study sample was in the instructional relativism stage, 23.10% were in interpersonal agreement, 35.76% were in social order and authority, 11.95% were in social contract and finally 3.80% were in universal ethical principles.

**Conclusion:** It was concluded and identified that the stages of moral judgement among the study sample indicate that interpersonal agreement, social order and authority are the most developed among university students.

## Introduction

When discussing moral judgement, it is essential to talk about “ethics”. This term refers to the fact that a person's behavior within society is regulated by rules. From childhood, human beings are immersed in socializing agents such as the family, the school, the environment, among others ( Mendizabal *et al.,* 2021). Thus, from an early age, children learn that there are both good and bad behaviors. All these opinions forged based on the different experiences and external agents about the moral problems they experience, and the arguments used in favor of these judgements constitute the development of the cognitive behavior of moral judgement ( Noguera, 2018).

As they pass through stages such as adolescence, youth and adulthood, individuals form their own identity, and become consciously aware of assuming and respecting norms and values. Lawrence Kohlberg (1973) points out that the person will learn and move up in the process of moral judgement in accordance with cognitive development. That is, as the person passes through various stages, he or she will find himself or herself at a different moral level, which will modify his or her reasoning ( Mendizabal *et al.,* 2021).

Kohlberg (1969) considers that the functioning of moral judgement is organized in cognitive structures from which all types of moral reasoning derive, which is based on universal and abstract principles, therefore, moral judgement is an evaluation and justification of prescriptive values versus what is good and bad ( Goenaga, Lopera & Villada, 2021).

Kohlberg organized the theory of moral development on the basis of three levels, pre-conventional, conventional and post-conventional. These are characterized by moral problems which, in turn, are divided into two stages representing the individual's criteria for behavior ( Cruz Puerto, 2020). The first level, the preconventional level, indicates that people are not guided by society's norms but only by concrete consequences; at the conventional level, the person adheres to society's norms and strives to avoid being punished; finally, at the postconventional level, the person reasons based on ethical principles beyond society's norms ( Sandoval, Villega & Vega, 2019).

At the beginning of university life, cognitive development is reinforced by teachers, who must contribute to the development of students' moral judgement as part of a comprehensive education ( Mendizabal *et al.,* 2021). Although programs already include ethics and moral issues in their curricula to guide them during their professional practice, the results are mostly imperceptible. Therefore, it is important that based on a diagnosis of the student's reasoning and moral development, the current educational purposes are evaluated and improved ( Contreras *et al.*, 2020).

Moral development has been considered an essential objective in education systems, as it is a progressive and gradual process that requires a combination of academic training, practical skills, and moral competences to be developed ( Ranjbar *et al.*, 2017). This is vital as previous studies show that there is an “ethical erosion” in higher education ( Reyes *et al.*, 2021), therefore, educational commitment must be addressed in the long term, where a theme of values, democracy, commitment, and justice is instilled as an aspect of lifelong learning ( Sandoval, Villega & Vega, 2019).

We must remember that educational institutions are an important partner in the moral development of individuals and society, facilitating change not only at the personal level, but also contributing to the community and society. Citizens must be educated in democratic values, where they exercise their rights in a responsible manner and fulfil their obligations in solidarity ( Pedraza & Pérez, 2019).

In view of this scenario, it was proposed as a general objective to identify and describe the stages of moral judgment among university students in the tenth semester in the district of Ica, in 2022 in a way that allows to demonstrate and respond to the problem that mobilized the study, that is, how are the levels of moral judgment in university students in the tenth semester in Ica in 2022?

For which the following specific objectives were set out: to identify moral judgement at the preconventional level, the conventional level, and the post-conventional level in university students in the tenth semester in Ica, in 2022.

## Methods

### Study design

The deductive research approach was used, because a general topic was analyzed and delimited to the particular and descriptive ( Ñaupas *et al*., 2018).

The study design was quantitative, considering the use of numerical scales for the development of results, non-experimental-observational, because the study was not conducted in a laboratory and the researcher did not intervene in the variables, and cross-sectional ( Hernández-Sampieri & Mendoza, 2018).

### Participants

The population included 265 university students in the tenth cycle of the administrative sciences career of the Universidad Nacional San Luis Gonzaga, Universidad Autonoma de Ica, Universidad Privada San Juan Bautista and Universidad Tecnologica del Perú, these universities are located in the district of Ica and are active and licensed by the Peruvian government. The type of sampling was non-probabilistic, according to Hernández-Sampieri & Mendoza (2018), indicating that this is done to a “subgroup of the population in which the choice of the elements does not depend on probability but on the characteristics of the research” and in turn by convenience, which, according to Valderrama (2014), indicates that the types of sampling by convenience or intentional “is characterized by a deliberate effort to obtain representative samples through the inclusion in the sample of typical groups”.

Given the above in the population, a total study sample of 157 university students (men and women), who were in the tenth cycle of the administrative sciences career, was considered, this being a representative number within the study. The total use of data from the proposed sample was made, not discarding, segregating or excluding any particular data.

### Instruments

The technique used was a survey and the instrument was the questionnaire called “Defining Issues Test” (DIT) which was developed by Rest (1979) and then translated into Spanish by Pérez (1997) as “Cuestionario de Problemas Sociomorales”. Then, Palacios (2003) made a manual called “El uso informatizado del cuestionario de problemas sociomorales (DIT) del Rest” and it was validated by Huanccollucho (2017) with a Cronbach's Alpha reliability of 0.70. The questionnaire was based on the theory of Lawrence Kohlberg (1973), which proposes to examine the moral judgement of a person in each period throughout his or her life and how this may evolve. The full questionnaire can be found in the Extended data.

Kholberg proposes six stages of moral judgement divided into three different levels of development ( Table 1).

**Table 1.** Stages of moral judgement.

Categories	Moral Judgement Stage
Preconventional	Obedience and punishment
	Self-interest
Conventional	Interpersonal accord and conformity
	Authority and maintaining social order
Postconventional	Social contract
	Universal ethical principles

The instrument is divided into six different stories (see Table 2), which are called “dilemmas”, where a short story on a specific topic is presented. Then, twelve items are posed, including questions and statements, per story.

**Table 2.** Stories and items.

Dilemmas	Questions and/or statements
Story 1: Henry and the medicine	12 items
Story 2: The occupation of the students	12 items
Story 3: The escaped prisoner	12 items
Story 4: The doctor's dilemma	12 items
Story 5: Mr. Gómez	12 items
Story 6: The magazine	12 items

At the end of each story, an importance chart is displayed (see Table 3), where the respondent must place the items that he/she considers most important, where 1 is the most important item, according to the student's criteria, and 4 is the item that generates the least importance for the person or the person he/she considers most important.

**Table 3.** Table of importance.

Rank in order of importance				
	1	2	3	4
N° de item				

Table 3 describes what was used to measure the level and development of moral judgement of each university respondent.

### Validation and reliability

The instrument was validated in 2022 to demonstrate its validity. This evaluation was carried out based on the judgment of two experts in the field of education and one in educational psychology, who analyzed, assessed and validated the data collection tool in order to determine whether the questions were pertinent, relevant and clear.

After obtaining the validation of the experts, the reliability of the instrument was determined by means of a pilot test with 20 people. The statistical reliability test used was Cronbach’s Alpha (α), which must be greater than 0.7 to indicate that the instrument is reliable. The value obtained from the test was 0.820, this result determined that the instrument is still reliable for its application. It is important to indicate that the people surveyed in the pilot test were not considered within the final sample.

### Procedures

The questionnaire survey was carried out using the Google Forms ^TM^ platform, which was distributed to each student in the tenth semester of the administrative sciences course, including instructions for correct completion. All this was done virtually, due to the wave of COVID-19 infection in Peru. The duration of data collection was from June 7 to June 14, 2022.

The statistical software SPSS version 25 was used for data analyzed and the Microsoft Excel 2019 program was used for all the data processing carried out to create tables with the results found. Regarding the presentation of the results of the article, this was done in a descriptive manner, which allowed us to identify the prevalence of the indicators of the moral judgement variable in the study sample. The data processing was carried out from June 15 to June 30, 2022.

### Ethical approval

The project was approved by the president of the ethics committee of the Universidad Autónoma de Ica by means of certificate CO-001-16-2022/CE issued on 15 February 2022.

### Consent

Participants in this study were of legal age according to the laws of the Peruvian state and did not need parental consent to complete the survey. Each of the students received a consent form containing all the information related to the development of the questionnaire, which they read, signed and then returned to the researchers, meaning that written informed consent was obtained from all participants.

The rights of the participants were developed based on three pillars, which were: First, confidentiality; this is because the information collected in the study was codified, therefore, all the answers obtained were processed under anonymity. Also, the participant's data and information were used exclusively for research purposes.

The second is beneficence; the research considered Helsinski’s ethical principles as the fundamental basis of the study, taking as a reference to avoid harm to third parties and even to the participant; for which the physical, psychological and social well-being of the participant was respected and sought at all times and finally, the principle of justice; since the participants of the study were treated fairly and equitably; providing the maximum possible protection and avoiding risks.

## Results

Demographic data showed that 54.14% of the university students analyzed were male and 45.86% were female. Regarding age, it was found that the vast majority of students were between the ages of 21 years (52.23%), followed by students aged 22 years (35.03%), also 23 years (10.83%) and finally 1.91% were 24 years or older (see Table 4).

**Table 4.** Demographic data of participants.

Demographic data	Items	N	%
Age	21 years	82	52.23%
	22 years	55	35.03%
	23 years	17	10.83%
	From 24 to more years	3	1.91%
Sex	Male	85	54.14%
	Female	72	45.86%
Total		157	100.0%

The preconventional level of the study sample was analyzed, where a development of instrumental relativism moral judgement of 12.75% is evident in the university students in their tenth semester of the administrative sciences (see Table 5). This result is exclusive of the evaluation of developmental stage II, because the test does not evaluate stage I.

**Table 5.** Moral judgement at the preconventional level.

Stage of moral judgement	N	%
Obedience and punishment	-	0.0
Self-interest	157	12.75
Total	157	12.75

N=number of participants.

We analyzed the conventional level where we find a development of moral judgement of interpersonal concordance of 23.10% and social order and authority of 35.76% in the university students in their tenth semester of the administrative sciences corresponding to development stage III and IV respectively (see Table 6).

**Table 6.** Moral judgement at the conventional level.

Stage of moral judgement	N	%
Interpersonal accord and conformity	157	23.10%
Authority and maintaining social order	157	35.76%
Total	157	58.86%

N=number of participants.

The post-conventional level was analyzed, showing a development of moral judgement of social contract of 11.95% and universal ethical principles of 3.80% in the university students in their tenth semester of the administrative sciences corresponding to developmental stage V and VI respectively, determining and reaching a post-conventional level of 15.75% among the study sample (see Table 7).

**Table 7.** Moral judgement at the post-conventional level.

Stage of moral judgement	N	%
Social contract	157	11.95%
Universal ethical principles	157	3.80%
Total	157	15.75%

N=number of participants.

Table 8 shows the A and M scores, which show the reliability of the data, and whether respondents have lied or had a bias when giving an answer. The scores should be less than 14%. If this is achieved, it means that the responses are truthful and do not need to be invalidated.

**Table 8.** Reliability of responses.

Stage of moral judgement	N	%
A	157	6.90%
M	157	5.70
Total	157	12.60%

N=number of participants.

**Figure 1.**
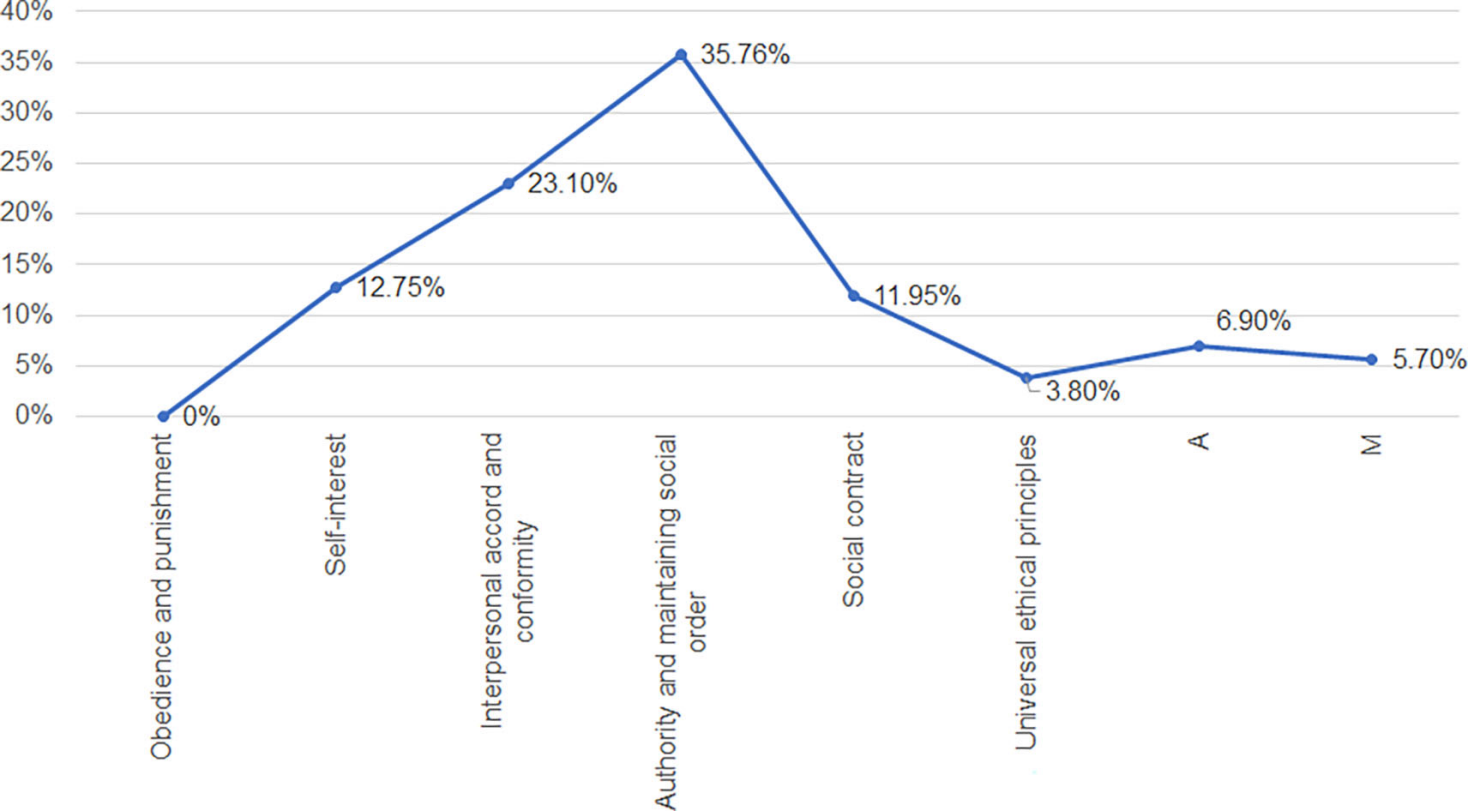
Moral judgement stage profile among the sample: General.

In reference to Figure 1, it is evident that the responses both A (6.90%) and M (5.70%) are less than 14%, so it takes as reliable all the responses collected by the students of the study site. Taking this into account, it is determined that the most predominant moral judgement among the students is the conventional one; being part of this the interpersonal agreement (23.10%) and social order and authority (35.76%): stage III and IV, indicating that the judgement goes with having good behaviors and behaving according to how others expect them to behave, also that they are governed by the regulations and laws determined and that they do not intend to infringe them intentionally, unless they are forced to break some law. Secondly, there is the post-conventional stage; being part of this the social contract (11.95%) and universal ethical principles (3.80%). This group has a judgement empathy, knowing and being aware that all people have different points of view, values, ways of life and so on. They are always being respectful of this, but not preventing that if they see or are in a situation in which they see that the basic and universal principles of a person are being affected; they will put these principles above anything else. And finally, there is the preconventional stage, being part of this the punishment-obedience (0.0%) and instrumental relativism (12.75%), this type of stage would indicate that people act only out of interest and when they know that they will get something beneficial for themselves and for the rest of the people involved, being fairness and justice their predominant ones.

## Discussion and conclusion

The moral judgment of tenth semester university students in the district of Ica was identified. The results showed a development of moral judgment in the “Self-interest” stage with 12.75%; an analysis of the “Obedience and punishment” stage was not made, since this is only analyzed in minors. In view of this, it was possible to identify that 12.75% of the total sample were in the preconventional stage, this being the level with the lowest frequency among the study sample. This result differs from that found by Juarez and Tananta (2020) because they found that most of the university students in their sample were at the preconventional level of moral judgment (58.75%), so it was determined that the determining moral judgment among the students was preconventional. It should be noted that at this level the moral sense and judgment is assumed in such a way that the norms are accepted and if they favor one’s own interests. This is due to the fact that the individual proposes to do what satisfies his interests, considering it fair that others also pursue theirs.

Secondly, it was identified that 23.10% of the development of moral judgment of university students are in the stage “Interpersonal agreement and conformity” and 35.76% were in “social order and authority”. These results indicated that 58.86% of the total sample were in the conventional stage, this being the level with the highest frequency among the study sample. The result is similar to what was found by Oporto (2018) who found that 66% of university students in the fourth year of the education and psychology career at a university in Arequipa-Peru showed to be in the conventional moral development level. At this level, the person identifies with the group and they try to live up to their family’s expectations. It is perceived as good and bad, according to how society represents it. At this level, the majority of young people and adolescents (of both sexes) are at this level, where they recognize social rules and the interests of others, in that sense the morality of the individual is linked to the personal and social relationships they have established but also emphasizes especially the rules of authority, considers it essential to maintain social order and is justified by an obligation of conscience that requires people to fulfill the obligations they have to society so it would be important to be considered as a “good person”, loyal, respectable, cooperative and pleasant.

There was also evidence of a development of moral judgment of the social contract of 11.95% and of universal ethical principles of 3.80%. These results indicated that a total of 15.75% of university students were in the post-conventional stage of development, this being the second most prevalent among the study sample. These results are supported by Bedoya *et al.* (2021) whose study found that the postconventional index was the second predetermining moral development among tenth cycle students of a university in the city of Manizales with a marguen of 29%, the first being the conventional level (50%). At this level the meaning of ethics for the human being is defined in terms of more abstract principles and values. The person who reaches this level defines and considers that some norms are unjust and could be changed or repealed. This indicates that there are students who have reached the highest stage, who no longer think only of themselves and of society, but who carry out an analysis where they can demonstrate that there are supreme values and rights that every society must guarantee, as well as universal moral principles that are different from the laws themselves.

We must remember that stage IV is characterized by the fact that the values of law and life are in conflict and people have problems when choosing between these two, in some cases this leads to see this stage (post-conventional) as inadequate to resolve situations in a society whose legal system denies basic human rights; where our behavior does not necessarily follow the dictates of our moral judgments, situations faced by students who have passed to this level of moral judgment.

The determining moral judgment identified among the tenth semester university students of the district of Ica was the conventional moral judgment with a total of 58.86% of the entire sample, followed by the post-conventional level (15.75%) and finally the preconventional (12.75%). This general analysis is supported by both Jaime (2019) where conventional moral reasoning also prevailed, with 68.8%. Also in Barreto (2018) who evidenced that university students in his sample were mostly at the conventional developmental level (51%). Oporto (2018) substantiates that university students being at this stage is normal, since “at this level most adolescents and young adults are found, so that social norms and the expectations of others, especially authority, are appreciated, and the subject identifies with the social role he or she occupies” (p. 22).

In all the cases reviewed, it is common for the populations and samples studied to be situated at the conventional level. This means that nowadays young people have become aware of abiding by the common rules of coexistence as well as recognizing that all individuals have interests that may not necessarily be the same. It follows that fairness is relative, as it is linked to personal interests, and that an exchange with others is necessary to ensure that one's own interests are satisfied.

It is important that, in the creation of scenarios for moral development, the educational and teacher's responsibility involves creating a type of conflict that facilitates the development of models of thinking in their students, because, as Kohlberg indicates, models of thinking are not taught, whereas, on the contrary, moral reasoning is self-generated in the environmental exchange and changes progressively.

The main limitation is the collection of data in a single period and not longitudinally to be able to observe how the stages change according to the knowledge and maturity of each person. Therefore, it is suggested that when replicating the studies, they can be carried out under the type of research mentioned above and thus observe the evolution of moral judgement.

This empirical work was intended to discover, describe and understand the level of moral development that, following Lawrence Kohlberg’s contributions, characterises a group of students. The results of this research provide evidence of which of the stages is the most developed by students who are about to finish their careers and go out into the workplace, which requires workers who perform through correct behaviour and currently these often feel shame, guilt or, on the contrary, feel organisational pride. In this way, it will allow universities to improve their educational models that favour student moral development as a way to improve academic and work performance, which requires changes in the current forms of interaction between teachers, students and study centre.

As a final conclusion, it was identified that the stages of moral judgement among the study sample indicate that interpersonal agreement (23.10%); and social order and authority (35.76%) are the most developed, instrumental relativism (12.75%) is the least developed and social contract (11.95%) and universal ethical principles (3.80%) are the stages that tend to develop in the future among university students in the tenth semester in the district of Ica.

Regarding the specific objectives, it was possible to identify that the preconventional level was 12.75%, the conventional level was 58.86% and finally the post-conventional level was 15.75% among university students.

Although the transition through each of the stages proposed by Kolhberg evolves over the years, one of the keys provided by Kolhberg’s theory to reach the last stage is the need to dialogue or contact with people or reflections in stages higher than ours to discover how they reason in situations of ethical conflict, It is therefore important that after the stages are located, the educational sector can carry out activities that reinforce the development of moral judgement and continue in ascending progression, but also that it transmits the capacity to live in accordance with this judgement, as many times there are not intellectual errors but moral weakness, due to a lack of strength to do good. This is because our moral action often shows incoherence with respect to our way of thinking, which forces us to re-evaluate the values we hold.

## Data Availability

Zenodo. Moral judgement in university students in Ica: a view from the perspective of L. Kohlberg Vr.4. https://zenodo.org/record/7199915#.Y0Nc-nZBy3A ( Pozo Enciso *et al.*, 2022). This project contains the following underlying data:
• SPSS – Moral judgement database.csv • SPSS – Moral judgement database.sav • MORAL JUDGEMENT DATA BASE.xlsx • MORAL JUDGEMENT DATA KEY.txt • MORAL JUDGEMENT PART 2.xlsx Zenodo. Moral judgement in university students in Ica: a view from the perspective of L. Kohlberg Vr.4. https://zenodo.org/record/7199915#.Y0Nc-nZBy3A ( Pozo Enciso *et al.*, 2022). This project contains the following extended data:
•
Figure 1 - Moral judgement stage profile among the sample: General • Social-moral problems questionnaire.docx Data are available under the terms of the Creative Commons Attribution 4.0 International license (CC-BY 4.0).
