# Supplementary material for: Moral judgement among university students in Ica: A view from the perspective of Lawrence Kohlberg
Source: F1000Res. 2023 May 9;11:1428. Originally published 2022 Dec 5. [Version 2] doi: 10.12688/f1000research.125433.2 (PMC10186059; doi:10.12688/f1000research.125433.2)
Supplement: Peer review history

# Reviewer report: Reviewer response for version 2

**DOI:** 10.5256/f1000research.146570.r172518

**Published:** 2025-08-19

**Authors:** Ebtsam Aly Abou Hashish

**Affiliations:** 1 Nursing Administration Department, Faculty of Nursing, Alexandria University, Alexandria, Egypt

**Title:** Moral judgement among university students in Ica: A view from the perspective of Lawrence Kohlberg

**Overall assessment**

 The manuscript examines the moral judgment levels of university students in their final semester, using Kohlberg’s theory and the Defining Issues Test (DIT). The topic is important for understanding moral development in higher education, and the study provides useful descriptive data for the Peruvian context. The revised version has improved methodological clarity, inclusion of demographic details, and a more updated discussion. However, further refinement is needed in theoretical justification, discussion depth, and implications for practice.

 1. Significance and originality

**Strengths**

- Addresses an important area of moral development in higher education.
- Applies Kohlberg’s theory systematically and uses a validated instrument (DIT).
- Adds local Peruvian data to a field where national-level evidence is limited.

**Areas for improvement**

- Clarify the study’s theoretical contribution beyond presenting descriptive prevalence data.
- Explicitly discuss why understanding moral judgment levels at this stage of study is important for educational policy, curriculum development, and professional ethics.
- Position the study more clearly within international moral development research.

2. Methodology

**Strengths**

- Quantitative, descriptive, cross-sectional design is clearly stated.
- Sampling method (non-probabilistic, convenience) and participant demographics are now described.
- Ethical approval and informed consent are documented.

**Areas for improvement**

- Provide a stronger justification for the chosen sample size and its representativeness.
- Expand on the validation process—specifically how the expert judgment ensured content validity and whether any cultural adaptation of the DIT was necessary.
- Describe more clearly how scoring and stage classification from the DIT were conducted.

3. Data analysis

**Strengths**

- Descriptive statistics are presented clearly with supporting tables.
- Reliability (A and M scores) is addressed, ensuring data credibility.

**Areas for improvement**

- Although descriptive analysis is appropriate for the study aim, integrating more comparative or subgroup analysis (e.g., by gender, age) could enrich the findings.
- Consider reporting confidence intervals for prevalence percentages to provide a sense of precision.

4. Results presentation

**Strengths**

- Tables are clear, with percentages and participant numbers.
- Logical organization by Kohlberg’s levels and stages.

**Areas for improvement**

- The narrative could better synthesize key patterns across levels, rather than repeating table data.
- Highlight notable differences between stages and their possible educational significance.

5. Discussion

**Strengths**

- Incorporates both local and international references.
- Compares findings with prior studies in Peru and abroad.

**Areas for improvement**

- Discussion remains mostly descriptive; provide deeper interpretation of why the conventional level dominates, linking to sociocultural or educational factors in Peru.
- Explicitly address implications for curriculum design, teaching methods, and student ethical development.
- Use more recent references (last 5 years) to strengthen relevance.
- Address the potential influence of study field (administrative sciences) on moral reasoning levels.

6. Limitations

**Strengths**

- Notes the limitation of cross-sectional design.

**Areas for improvement**

- Discuss limitations of using self-report surveys for moral judgment assessment.
- Acknowledge that findings may not generalize beyond the specific universities or fields studied.

7. Language and clarity

- Overall clear and readable, but some sentences in introduction and discussion are lengthy and could be more concise.
- Ensure consistency in terminology (e.g., “moral judgement” vs. “moral judgment”).
- Minor grammar and style edits would improve flow.

**Recommendation:**

**Minor to moderate revision** – The study is sound and provides useful descriptive data, but should be strengthened by clarifying theoretical contribution, deepening discussion, and articulating practical implications.

**Key recommendations summary:**

1. Strengthen theoretical rationale and contribution to moral development literature.
2. Expand discussion with sociocultural interpretation and educational implications.
3. Update and integrate more recent references.
4. Provide greater detail on DIT scoring and validation.
5. Improve clarity and conciseness in presentation.

Is the work clearly and accurately presented and does it cite the current literature?

Partly

If applicable, is the statistical analysis and its interpretation appropriate?

Yes

Are all the source data underlying the results available to ensure full reproducibility?

Yes

Is the study design appropriate and is the work technically sound?

Yes

Are the conclusions drawn adequately supported by the results?

Partly

Are sufficient details of methods and analysis provided to allow replication by others?

Yes

Reviewer Expertise:

education- research- management - leadership - ethics - quality of care

I confirm that I have read this submission and believe that I have an appropriate level of expertise to confirm that it is of an acceptable scientific standard, however I have significant reservations, as outlined above.

# Reviewer report: Reviewer response for version 2

**DOI:** 10.5256/f1000research.146570.r172517

**Published:** 2023-05-25

**Authors:** Vered Ne’eman-Haviv

**Affiliations:** 1 Department of Criminology, Ariel University, Ariel, Israel

I thank the authors for answering the comments. I have no further comments.

Is the work clearly and accurately presented and does it cite the current literature?

Yes

If applicable, is the statistical analysis and its interpretation appropriate?

Yes

Are all the source data underlying the results available to ensure full reproducibility?

Yes

Is the study design appropriate and is the work technically sound?

Yes

Are the conclusions drawn adequately supported by the results?

Partly

Are sufficient details of methods and analysis provided to allow replication by others?

Yes

Reviewer Expertise:

morality, drug use

I confirm that I have read this submission and believe that I have an appropriate level of expertise to confirm that it is of an acceptable scientific standard.

# Reviewer report: Reviewer response for version 2

**DOI:** 10.5256/f1000research.146570.r172516

**Published:** 2023-05-15

**Authors:** Ambrocio Teodoro Esteves Pairazamán

**Affiliations:** 1 Facultad de Farmacia y Bioquímica, Universidad Privada Norbert Wiener, Lima, Peru

The work seems to me to be much more complete in this second version, where I can see that the discussion of results with previous international and local antecedents have been improved.

Is the work clearly and accurately presented and does it cite the current literature?

Yes

If applicable, is the statistical analysis and its interpretation appropriate?

Yes

Are all the source data underlying the results available to ensure full reproducibility?

Yes

Is the study design appropriate and is the work technically sound?

Yes

Are the conclusions drawn adequately supported by the results?

Partly

Are sufficient details of methods and analysis provided to allow replication by others?

Yes

Reviewer Expertise:

Master's degree in Research and Teaching, and a doctorate in Educational Administration.

I confirm that I have read this submission and believe that I have an appropriate level of expertise to confirm that it is of an acceptable scientific standard.

# Reviewer report: Reviewer response for version 1

**DOI:** 10.5256/f1000research.137736.r165585

**Published:** 2023-04-24

**Authors:** Ambrocio Teodoro Esteves Pairazamán

**Affiliations:** 1 Facultad de Farmacia y Bioquímica, Universidad Privada Norbert Wiener, Lima, Peru

The discussion of results should be more direct and concise. Above all, each result found should be discussed with previous studies related to the research topic, showing whether the results are similar or different, either in the same country where the study was conducted or in another part of the world. It is recommended to give a brief analysis of the findings. Preferably the studies intended for discussion should not be older than 5 years.

Is the work clearly and accurately presented and does it cite the current literature?

Yes

If applicable, is the statistical analysis and its interpretation appropriate?

Yes

Are all the source data underlying the results available to ensure full reproducibility?

Yes

Is the study design appropriate and is the work technically sound?

Yes

Are the conclusions drawn adequately supported by the results?

Partly

Are sufficient details of methods and analysis provided to allow replication by others?

Yes

Reviewer Expertise:

Master's degree in Research and Teaching, and a doctorate in Educational Administration.

I confirm that I have read this submission and believe that I have an appropriate level of expertise to confirm that it is of an acceptable scientific standard, however I have significant reservations, as outlined above.

# Reviewer report: Reviewer response for version 1

**DOI:** 10.5256/f1000research.137736.r165032

**Published:** 2023-03-07

**Authors:** Vered Ne’eman-Haviv

**Affiliations:** 1 Department of Criminology, Ariel University, Ariel, Israel

The article is well written, clearly and concisely.

 The main problem with the article is that its theoretical contribution and the rationale for its execution are not clear. What does the knowledge about the moral level of the students contribute to us? The study did not examine the correlation of the level of morality with other variables, but only presents descriptive information about their level of morality, and therefore its contribution to existing research knowledge is not clear.

 Beyond that, there is a lack of data about the subjects: how many men and how many women", at what age? etc. There is no significant information about them.

Is the work clearly and accurately presented and does it cite the current literature?

Yes

If applicable, is the statistical analysis and its interpretation appropriate?

Yes

Are all the source data underlying the results available to ensure full reproducibility?

Yes

Is the study design appropriate and is the work technically sound?

Yes

Are the conclusions drawn adequately supported by the results?

Partly

Are sufficient details of methods and analysis provided to allow replication by others?

Yes

Reviewer Expertise:

morality, drug use

I confirm that I have read this submission and believe that I have an appropriate level of expertise to confirm that it is of an acceptable scientific standard, however I have significant reservations, as outlined above.

# Reviewer report: Reviewer response for version 1

**DOI:** 10.5256/f1000research.137736.r159291

**Published:** 2023-02-03

**Authors:** Ebtsam Aly Abou Hashish

**Affiliations:** 1 Nursing Administration Department, Faculty of Nursing, Alexandria University, Alexandria, Egypt

The paper is clear and comprehensive. I need the authors to describe the target population and their number because the sample is considered a relatively small one. Explain in detail how you ensured the validity and dependability of tools. In addition, how did you maintain the human rights of your participants?

 Your discussion needs to be more direct, and you need to discuss each finding with related supportive or opposing prior studies. Add direct implication to improve students' moral judgment.

 Your references need to be updated, especially in the discussion section, to be within the last five years.

Is the work clearly and accurately presented and does it cite the current literature?

Partly

If applicable, is the statistical analysis and its interpretation appropriate?

Yes

Are all the source data underlying the results available to ensure full reproducibility?

Yes

Is the study design appropriate and is the work technically sound?

Yes

Are the conclusions drawn adequately supported by the results?

Partly

Are sufficient details of methods and analysis provided to allow replication by others?

Yes

Reviewer Expertise:

education- research- management - leadership - ethics - quality of care

I confirm that I have read this submission and believe that I have an appropriate level of expertise to confirm that it is of an acceptable scientific standard, however I have significant reservations, as outlined above.

## References

[1] : Academic Ethical Awareness and Moral Sensitivity of Undergraduate Nursing Students: Assessment and Influencing Factors. *SAGE Open Nurs* .2021;7: 10.1177/23779608211026715 23779608211026715 10.1177/23779608211026715 34263030 PMC8243134
